# Evaluating a Clinical Decision Support System for Optimizing Total Parenteral Nutrition in Adult Oncology Patients

**DOI:** 10.3390/nu18040640

**Published:** 2026-02-15

**Authors:** Alexandra Foscolou, Christina Kostara, Aristea Gioxari

**Affiliations:** 1 Department of Nutritional Science and Dietetics, School of Health Sciences, 24100 Kalamata, Greece; alexandra.foscolou@go.uop.gr; 2 Department of Nutrition, IASO Hospital, 37-39 Kifissias Ave., 15123 Athens, Greece; chistina@cxristinakostara.gr

**Keywords:** parenteral nutrition, parenteral nutrition prescription, ready-to-use parenteral solutions, clinical decision support system, oncology, adults

## Abstract

Background/Objectives: In oncology patients, total parenteral nutrition (TPN) prescriptions are complex and depend on individual nutritional and clinical status. Prescriptions for TPN solutions often result in over- or under-dosing of specific nutrients, due to the large number of ingredients and formulation-related constraints. Clinical support decision systems (CDSSs) may assist clinicians in complex decision-making processes. The aim of this study was to quantify discrepancies between CDSS TPN prescriptions and ready-to-use PN formulation compositions administered in routine clinical practice. Methods: In this cross-sectional study, 40 hospitalized adult oncology patients who were prescribed TPN were recruited. CDSS data was used to calculate individual nutritional needs (i.e., fluids, micro-/macronutrients, and electrolytes) and utilized to identify the closest available standardized ready-to-use TPN formula corresponding to those needs. The algorithm created by the use of the CDSS was based on evidence-based equations from ASPEN. We compared the CDSS-calculated nutritional needs with the ready-to-use TPN formula and identified nutrients that had to be added to the TPN solution bag. Results: The daily needs of all macronutrients were fully covered by the ready-to-use TPN, while actual intake of micronutrients, except for phosphorus (P), was insufficient and had to be supplemented in the TPN bag (amino acids: +21.1%, *p* < 0.001; lipids: +8.4%, *p* = 0.023; P: +32.5% *p* = 0.001; Na: −30.5%, *p* < 0.001; K: −50.6% (*p* < 0.001); Ca: −51.7%, *p* < 0.001). Conclusions: The use of the CDSS tool could facilitate PN prescriptions by indicating the most suitable standardized commercial PN solutions to support patients’ nutritional needs and help physicians assess the patient’s additional needs.

## 1. Introduction

Parenteral nutrition (PN) is a key therapeutic tool for patients who cannot meet their nutritional needs orally or enterally [1]. Unlike enteral nutrition, which requires a functional gastrointestinal tract, PN completely bypasses the intestine, delivering nutrients directly into the bloodstream via intravenous administration [2]. Parenteral nutrition is widely used in patients with severe gastrointestinal disorders and in patients with cancer, where the adequate and safe administration of energy, protein, and other nutrients is directly associated with the outcome of the disease [3,4]. Recent evidence underscores the complexity of nutritional management in oncology and the necessity for systematic, individualized strategies to support appropriate enteral or parenteral nutrition decisions [5,6]. Despite its importance, PN is a particularly complex and potentially high-risk intervention, as it requires precise calculations, the careful selection of formulations, and the close monitoring of metabolic parameters [7].

Ideally, the prescription for PN should be based on an individualized assessment of the patient’s needs for energy, amino acids, carbohydrates, fat, electrolytes, trace elements and fluids. However, translating these theoretical or “ideal” needs into an actual solution that will be prepared and administered to the patient is not a straightforward process [8]. Multiple and complex stages are involved that may alter prescribed versus actual nutrient delivery. These stages include medical prescription, processing by the dietitian and clinical pharmacist, restrictions imposed by commercially available formulations or ready-to-use bags, pharmacy practices during compounding, and, finally, actual administration [8,9]. At each one of these stages, errors and deliberate modifications may occur. Recording or calculation errors, incorrect formulation selection, rounding, adjustments due to volume or osmolarity restrictions, and product availability issues can lead to a final solution with significant deviations from what was originally prescribed [10,11,12]. This creates a practical gap between the “prescribed” and the actual PN solution administered, which often remains invisible in everyday clinical practice.

These inconsistencies may have clinical consequences. Underestimating energy or protein intake contributes to worsening nutritional status, while overestimating can lead to overeating, hyperglycemia, liver and hepatic dysfunction, and respiratory stress [13,14]. Similarly, discrepancies in electrolyte and fluid administration increase the risk of metabolic and cardiorespiratory complications [15]. Nevertheless, the systematic recording and quantification of differences between the prescription and the final administered PN mixture are limited, and PN quality assessment often focuses exclusively on the accuracy of the initial prescription. Individually tailored PN regimens should be preferred over standardized PN solutions when the nutritional requirements do not meet the available range of standard PN formulations [16]. As recommended by the ESPEN society, computerized prescriptions, whether standardized or individualized, should be used in the PN ordering process [16]. Recently, as part of the broader digitization of healthcare services, clinical decision support systems (CDSSs) have been developed and integrated into nutritional prescription procedures [17,18]. Recent technology has enabled the development of CDSSs that assist clinicians in complex decision-making processes based on evidence-based practices [19,20,21]. Regarding PN, such systems provide easy-to-use methods for the accurate calculation of nutritional needs. In addition, electronic ordering systems can still allow individualization of PN prescriptions, thus improving biochemical control and decreasing wastage [22].

CDSS have already been evaluated in randomized clinical trials, where they have demonstrated accuracy in calculating nutritional needs and clinical effectiveness in different populations [17,18,23]. However, it remains unknown whether personalized recommendations can be applied in practice to oncology patients when PN is administered exclusively in ready-to-use formulations. Due to disease-related metabolic alterations, treatment side effects, and reduced appetite, oncology patients are more prone to experiencing malnutrition [24]. Malnutrition is common in oncological patients, impacting a large percentage of this population. It may lead to considerable weight loss, reduced therapeutic effectiveness and worse health-related quality of life [25,26]. Thus, the aim of the present work was to quantify discrepancies between CDSS-calculated individualized TPN prescriptions and ready-to-use PN formulations administered in routine clinical practice. By quantifying the discrepancies in macro- and micronutrients, we aim to highlight the “personalization gap” that may exist between theoretically optimal and applied parenteral therapy.

## 2. Materials and Methods

### 2.1. Study Design

An observational, cross-sectional study was conducted on oncology patients receiving total PN (TPN) during their hospitalization at IASO Hospital in Athens, Greece.

### 2.2. Sample

The study included all eligible, hospitalized oncological patients (>18 years) of both sexes, who received TPN formulations during the period January 2023–December 2025. All individuals were patients at the IASO Hospital in Athens, Greece. More specifically, the study included hospitalized adult oncology patients with gastrointestinal malignancies, including esophageal, gastric, pancreatic, colorectal, and biliary tract cancers, as well as cases with peritoneal carcinomatosis. TPN was initiated when oral or enteral nutrition was not feasible or adequate, mainly due to postoperative gastrointestinal dysfunction following major abdominal surgery, malignant bowel obstruction, prolonged ileus, high-output fistulas, severe gastrointestinal intolerance related to anticancer treatment, or severe disease-related malnutrition. Patients were recruited for the study on the day that they started receiving TPN support.

### 2.3. Inclusion and Exclusion Criteria

Inclusion criteria:

- Adults (>18 years);
- Gastrointestinal malignancy diagnosis;
- Already receiving chemotherapy at the time of PN;
- Administration of standardized ready-to-use TPN formulations for at least 24 h.

Exclusion criteria:

- Receiving individualized TPN solutions.

### 2.4. Bioethics

The Ethics Committee of IASO HOSPITAL in Athens, Greece, examined and approved the study (Approval Code 21-1118G). The study was carried out in accordance with the principles of the Helsinki Declaration (1989) and its later amendments. Participants were informed of the study aims, and written informed consent was provided prior to enrollment.

### 2.5. Measurements

Data collection was performed by the medical and nursing staff of the IASO hospital. More specifically, we collected data regarding:

Sociodemographic characteristics: Data on age (years) and sex (male/female) were recorded.

Anthropometry: Weight (kg) and height (m) were measured using standard procedures, and body mass index (BMI) in kg/m^2^ was calculated. Normal weight was defined as a BMI between 18.5 and 24.9 kg/m^2^, underweight as a BMI < 18.5 kg/m^2^, overweight as a BMI between 25 and 29.9 kg/m^2^, and obesity as a BMI ≥ 30 kg/m^2^.

Blood measurements: Following an overnight fast before the beginning of the TPN, each patient supplied a morning whole blood sample (20 mL) for the separation of serum and plasma. All laboratory measurements were obtained at baseline, prior to the initiation of TPN, and were used for nutritional assessment and CDSS calculations. Ethylenediamine tetra-acetic acid (EDTA) was used for plasma isolation. For serum isolation, whole blood was previously allowed to clot at room temperature for 20 min. Whole blood samples were centrifuged at 3000 rpm for 10 min at 4 °C. Serum glucose (mg/dL), albumin (g/dL), Na (sodium) (mmol/L), K (potassium) (mmol/L), Ca (calcium) (mg/dL) and P (phosphorus) (mg/dL) were quantified with an automatic biochemical analyzer using the manufacturer’s reagents (Cobas 8000 modular analyzer, Roche Diagnostics GmbH, Mannheim, Germany)

**Figure 1:**
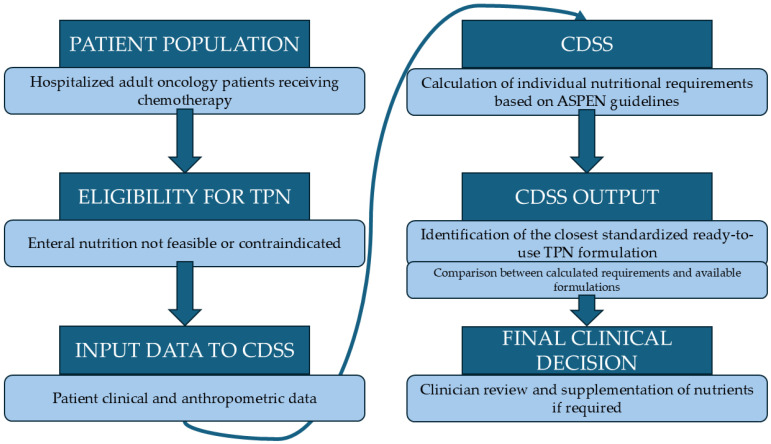
Workflow of CDSS-supported TPN in hospitalized adult oncology patients. TPN: total parenteral nutrition; CDSS: clinical decision support system.

Calculation of nutrient requirements: A CDSS (NutrinetPN, CibusMed, Athens, Greece) assessed each patient’s nutritional needs [23]. The specific CDSS is a clinically implemented decision-support system tool routinely used at IASO’s hospital practice. The CDSS was developed using a client–server architecture implemented in C# on the Microsoft.NET Framework 4.8, using Visual Studio 2019 and SQL Server 2019. The system incorporates an extensive library of age-specific PN protocols applicable to neonates, infants, children, adolescents and adults [23]. Briefly, the system uses evidence-based statistical models to determine nutritional parameters, evaluates input data (anthropometric data, comorbidities, clinical status, and specific treatment information), and computes individual daily nutritional requirements for energy, macronutrients, vitamins, and minerals, as well as the concentration of each nutrient and daily PN volume [23], all in accordance with the American Society for Parenteral and Enteral Nutrition (ASPEN) guidelines [27]. It does not autonomously prescribe PN but supports clinician decision-making based on guidelines. In case of absence of specific guidelines, parameter ranges are derived from the up-to-date literature. An internal database of commercially available TPN components enables the CDSS to assist clinicians in selecting appropriate solutions according to the prescriber’s input, facilitating the construction of complete PN formulations aligned with the clinician’s specified requirements. The composition of commercial TPN formulations was obtained from the official product specifications of the companies (Figure 1).

In the present study, the type and exact composition of the commercial ready-to-use TPN solutions that were ultimately administered were recorded. Ready-to-use PN formulations administered included commercially available two- and three-chamber PN bags providing standard macronutrients (amino acids, glucose, and lipids) and electrolytes. They do not routinely include specific immunonutrients (e.g., alanyl-glutamine dipeptides) or micronutrients, which are commonly administered separately due to stability constraints and the need for individualized dosing, in accordance with current clinical practice and guideline recommendations [28]. Commercially available pre-mixed PN bags were used in our study, reflecting routine clinical practice for PN in Greek hospitals. Some of the commercial PN formulations used [e.g., soybean oil + medium-chain triglycerides + olive oil + fish oil (SMOF)- or olive oil-based lipid emulsions] do contain lipid components with potential immunomodulatory properties, and these were preferentially selected by the CDSS in clinical scenarios associated with increased inflammatory burden or long-term PN tolerance.

For each patient, the regimen recommended by the CDSS was directly compared to the regimen of the commercial TPN solution that was ultimately administered.

### 2.6. Statistical Analysis

Categorical variables were presented as counts (n) and percentages (%), while the continuous variables were presented as means and standard deviations for normally distributed variables or as medians (IQR: interquartile range) for skewed variables (i.e., lipids). Normality was assessed using the Shapiro–Wilk test. Paired-sample *t*-test was used to identify differences between the two groups (i.e., CDSS TPN solution vs ready-to-use TPN bag) for normally distributed continuous variables and the Wilcoxon test for skewed variables. Statistical significance was set at *p* < 0.05. All statistical analyses were performed using the SPSS software, version 29.0 (IBM Statistics, Greece).

## 3. Results

Basic sociodemographic and anthropometrical characteristics of the N = 40 oncology patients receiving TPN are presented in Table 1. The study included both male (n = 11, 27.5%) and female (n = 29, 72.5%) patients. Overall, patients were of an older age (63.5 ± 12 years), had on average a BMI corresponding to normal weight status, and colorectal cancer was the most prevalent malignancy (32.5%).

In Table 2, baseline biochemical parameters of the N = 40 oncology patients are depicted. Glucose, Na, K, Ca and P were within the reference ranges, whereas serum albumin concentrations were below normal values.

In Table 3, comparisons between CDSS-derived TPN and ready-to-use TPN bags are presented. It was revealed that ready-to-use TPN bags had, on average, higher concentrations of amino acids (*p* < 0.001), lipids (*p* = 0.023) and phosphorus (*p* = 0.001), and lower concentrations of Na, K and Ca (all *p*’s < 0.001) compared to the TPN regimen derived from the CDSS. No other statistically significant differences were found between the two groups (all *p*’s > 0.05).

**Figure 2:**
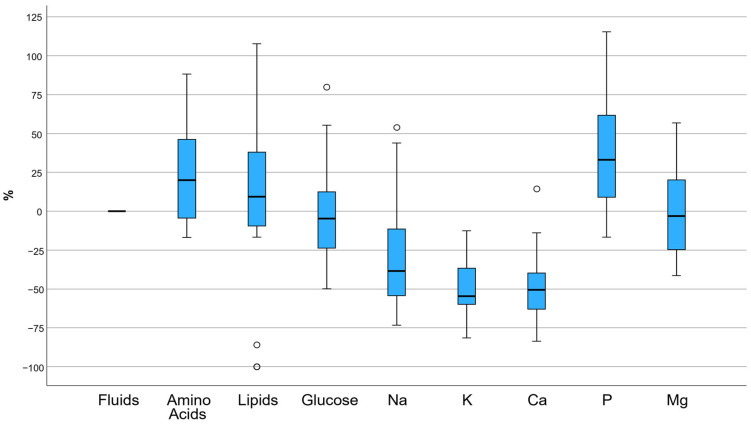
Boxplots of the percentage deviation between CDSS-derived TPN and administered via ready-to-use PN bags for each nutrient. Positive values indicate over-administration via the ready-to-use bags, while negative values indicate under-administration. The horizontal line within each box represents the median, boxes indicate the interquartile range, and whiskers represent the minimum and maximum values excluding outliers. Circles indicate outliers. Na: sodium; K: potassium; Ca: calcium; P: phosphorus; Mg: magnesium.

Further analyses revealed that amino acid, lipids and phosphorus provision showed mean percentage deviation of +21.1% (*p* < 0.001), +8.4% (*p* = 0.023) and +32.5% (*p* = 0.001), respectively, compared to the requirements derived by the CDSS, indicating significant variation between the recommended and administered PN. On the other hand, sodium, potassium and calcium provision showed mean percentage deviation of −30.5% (*p* < 0.001), −50.6% (*p* < 0.001) and −51.7% (*p* < 0.001), respectively (Figure 2).

## 4. Discussion

This observational study aimed to quantify discrepancies between CDSS-calculated individualized TPN prescriptions and the composition of ready-to-use PN formulations administered in routine clinical practice. The contribution of the present work does not lie in the evaluation of clinical adequacy of specific commercial TPN formulations used in clinical medicine, which is well established, but in demonstrating how a CDSS can support clinicians in matching individual patient requirements to available formulations and in determining the need for targeted nutrient supplementation. It was found that certain macro- and micronutrients, such as fluids, glucose and magnesium, showed no deviations between the CDSS-recommended and administered amounts, suggesting close alignments for these components. However, other components showed high positive or negative deviations. Amino acids, lipids and phosphorus were administered in higher amounts, whereas basic electrolytes such as sodium, potassium and calcium were administered in lower amounts. These findings may reflect the limitations of ready-to-use formulations, particularly for electrolytes, which require personalization according to the patient’s clinical profile and laboratory parameters. The added value of CDSS lies in supporting the standardization of TPN prescribing and reducing variability in clinical decisions, particularly in high-risk nutritional populations such as oncological patients. This digital tool may also help the prescribing physician in assessing the patient’s additional electrolyte needs and whether it is necessary to enrich the bag.

Our results are consistent with previous studies that have shown that standardized PN formulations are often unable to meet individualized needs [29], especially in high-risk populations such as oncology patients [7,30]. It has been shown that ready-to-use TPN bags are useful for standardization and reducing errors, but they lack flexibility if nutrient additions should be done [31]. The clinical significance of the observed deviations could be considered even greater given that the study was conducted on oncology adult patients, who often have increased metabolic demands [32], inflammation [33], and frequent electrolyte disturbances [34]. The observed discrepancies in several nutrients (i.e., amino acids, lipids, Na, K, Ca, and P) may be explained by the variability of oncology patients’ needs [35] combined with the inflexibility of premixed TPN solutions. Accurate coverage of nutritional needs in this patient group is critical, as even small deviations can affect treatment tolerance, response to chemotherapy, and overall prognosis [36]. The observed higher amino acid and fat intake could be considered as a burden for the metabolic health of a patient with already vulnerable renal function or elevated levels of inflammation [37,38]. In addition, it may also have implications for hepatic function, especially in prolonged PN administration [39,40]. Similarly, positive deviations in phosphorus could potentially pose a risk of hyperphosphatemia or metabolic complications, especially in patients with bone metastases or underlying renal insufficiency [41,42,43]. On the other hand, covering sodium, potassium, and calcium needs is often particularly important in oncology patients, as their needs may change due to gastrointestinal losses, chemotherapy regimens or dehydration [44,45]. The frequent occurrence of hypokalemia and hyponatremia in oncology patients [46] requires an individualized approach, which is difficult to achieve with fixed, ready-to-use TPN bags. Inadequate coverage of these electrolytes may lead to cardiac arrhythmias, neuromuscular weakness, increased fatigue, and acid–base balance disorders, events that can affect the course of treatment [47].

The use of CDSS has been shown to improve the accuracy of nutritional prescribing and reduce errors [23,48]. More specifically, the CDSS used in the present study has already been shown to be a facilitator for health professionals during PN prescription, since it can be considered as a supporting tool to determine the right nutrient concentrations by minimizing the risk of human error [23]. Moreover, the CDSS used in our study has already proven its clinical usefulness. In a randomized trial in pregnant women, its use led to greater adherence to the Mediterranean diet, a better nutritional status and a significant reduction in anxiety and depressive symptoms compared to the control group [48]. In addition, in another randomized controlled study of women with breast cancer, the same CDSS has been shown to improve adherence to the Mediterranean diet, reduce body weight, and enhance quality of life, supporting its usefulness in high-risk clinical populations [18]. Finally, in a relatively recently published randomized controlled trial of adolescents with polycystic ovary syndrome, the use of this CDSS led to a significant improvement in adherence to the Mediterranean diet, favorable changes in dietary profile, and reduced anxiety, supporting the role of this CDSS as an effective clinical management tool in high-risk endocrinology populations [17]. It should be noted that this CDSS and all related ones function as a decision-support tool designed to assist clinicians in aligning TPN prescriptions with guideline-based recommendations and not as a clinical gold standard. They do not replace clinical judgment, since it is the clinical team’s responsibility to prescribe decisions.

It is noteworthy that the effectiveness of CDSS tools in oncological patients has also been presented by other research groups. A deep learning-based CDSS developed for the detection and classification of gastric cancers showed high diagnostic performance in real-time clinical settings [49], further supporting the potential of CDSS technologies to assist clinicians in the management of oncology patients. These findings demonstrate that CDSS can enhance the quality of clinical practice by improving adherence to guidelines and therapeutic efficacy without increasing adverse events. However, the implementation of their recommendations remains limited when the available ready-to-use TPN formulas do not allow further additions. CDSSs could guide the physician to the most adequate standardized solution and optimize the use of individualized solutions, as well as keep a record of the patient’s nutritional profile.

To the best of our knowledge, this is one of the first studies quantifying whether ready-to-use PN solutions are aligned with CDSS-derived prescriptions in adult oncology patients in routine clinical practice. However, the current observational study has some limitations. The observational design does not allow causal inference. Future clinical intervention studies evaluating the efficacy of CDSS-prescribed TPN on anthropometrical, clinical and laboratory outcomes of oncology patients should be conducted. In such studies, patient characteristics, i.e., disease duration, chemotherapy regimens and TPN duration, should be recorded. Additionally, the CDSS of the present study was designed to support standard PN prescriptions and did not generate specific recommendations regarding adjunct immunonutrient supplementation. The lack of individualized guidance on immunonutrition represents a limitation of the current CDSS model. Future iterations could incorporate evidence-based recommendations on the appropriate indication and dosing of selected immunonutrients (e.g., glutamine or omega-3 fatty acids), particularly in oncology patients with defined clinical indications. The sample of the present cross-sectional study was relatively small (N = 40) and was limited to oncology patients of a specific hospital, which may limit the generalizability of the results. Moreover, the ready-to-use TPN bag formulations used, even though they were six different formulations, may differ in composition from those used in other hospitals or countries. Despite these limitations, the study clearly highlights the need for greater flexibility in the available PN formulations and the value of personalization, especially in high-risk populations such as oncology patients.

## 5. Conclusions

To conclude, the findings of the present work highlight significant discrepancies between personalized PN, as calculated by the CDSS, and the actual composition administered through standardized ready-to-use formulations. Although the CDSS incorporates the most updated guidelines and allows for the precise determination of each patient’s nutritional needs, the implementation of these recommendations is largely limited by availability and fixed, ready-to-use TPN bag formulations. The combination of a CDSS prescription and the use of multi-chamber PN bags may enhance the ability to rely on standardized PN with minimal usage of individualized prescriptions. Moreover, a possible practical approach would be to use CDSS not only to assist clinicians in aligning TPN prescriptions with guideline-based recommendations and defining nutritional requirements but also as a tool to support the selection of the most appropriate ready-to-use TPN formulation and help the prescribing physician assess the patient’s additional electrolyte needs. Improving the correspondence between prescribed and administered PN may have a significant impact on nutrition, recovery, and treatment tolerance in oncology patients, which should be further investigated using prospective cohort studies. Future prospective cohort studies using a broader range of formulations could also evaluate whether prescriptions derived from CDSSs may improve clinical outcomes, such as electrolyte stability and nutrition-related complications.

## Figures and Tables

**Table 1:** Sociodemographic and anthropometrical characteristics of the N = 40 oncology patients.

Characteristics	Mean ± Standard Deviation (or as Otherwise Stated)
Age (years)	63.5 ± 12 [range: 33–81]
Males, n (%)	11 (27.5)
Females, n (%)	29 (72.5)
Height (cm)	168 ± 8.5
Weight (kg)	58.7 ± 15
BMI (kg/m^2^)	20.9 ± 5.2
BMI Status	
Underweight, n (%)	10 (32)
Normal Weight, n (%)	17 (55)
Overweight, n (%)	2 (6.5)
Obese, n (%)	2 (6.5)
Cancer type	
Pancreatic cancer, n (%)	10 (25)
Colorectal cancer, n (%)	13 (32.5)
Gastric cancer, n (%)	7 (17.5)
Biliary tract cancer, n (%)	6 (15)
Peritoneal carcinomatosis, n (%)	3 (7.5)
Esophageal cancer, n (%)	1 (2.5)

Categorical values are presented as frequencies (%) and continuous as mean and standard deviation (SD). BMI: body mass index.

**Table 2:** Biochemical parameters of the N = 40 oncology patients.

Characteristics	Reference Range	Mean ± Standard Deviation
Albumin (g/dL)	3.5–5.5	3.4 ± 0.3
Glucose (mg/dL)	70–110	88 ± 8.0
Na (mmol/L)	136–145	139 ± 4.1
K (mmol/L)	3.5–5.1	4.1 ± 0.4
Ca (mg/dL)	8.4–10.2	9.1 ± 0.5
P (mg/dL)	2.3–4.7	3.3 ± 0.5

Results are presented as mean and standard deviation (SD). Na: sodium, K: potassium, Ca: calcium, P: phosphorus.

**Table 3:** Comparisons of CDSS-derived TPN regimens with administered ready-to-use TPN bags in the N = 40 oncology patients.

TPN Composition	TPN from CDSS	Ready-to-Use TPN Bags	*p*-Value
Fluids (ml)	1594 ± 489	1594 ± 489	1.00
Amino acids (g)	47 ± 15	59 ± 25	<0.001
Lipids (g)	50 (25)	54 ± 46	0.023
Glucose (g)	175 ± 70	161 ± 73	0.125
Na (mEq)	87 ± 41	54 ±21	<0.001
K (mEq)	84 ± 30	39 ± 14	<0.001
Ca (mEq)	16 ± 9.1	6.7 ± 2.2	<0.001
P (mmol)	14 ± 6.5	17 ± 5.9	0.001
Mg (mEq)	14 ± 6.5	12 ± 5.0	0.98

Values are presented as mean and standard deviation (SD) if normally distributed and median and IQR (interquartile range) for skewed variables (i.e., lipids). *p*-values derived from paired-sample *t*-test for normally distributed variables and the Wilcoxon test for skewed variables (i.e., lipids). CDSS: clinical decision support system; TPN: total parenteral nutrition; Na: sodium; K: potassium; Ca: calcium; P: phosphorus; Mg: magnesium. Statistical significance was set at *p*-value < 0.05.

## Data Availability

Data are unavailable due to privacy or ethical restrictions.

## Abbreviations

The following abbreviations are used in this manuscript:

BMI	Body Mass Index
CDSS	Clinical Decision Support System
Ca	Calcium
EDTA	Ethylenediamine tetra-acetic acid
K	Potassium
Mg	Magnesium
Na	Sodium
P	Phosphorus
PN	Parenteral Nutrition
TPN	Total Parenteral Nutrition
